# Hybridization speeds adaptive evolution in an eight-year field experiment

**DOI:** 10.1038/s41598-019-43119-4

**Published:** 2019-05-01

**Authors:** Nora Mitchell, Gregory L. Owens, Stephen M. Hovick, Loren H. Rieseberg, Kenneth D. Whitney

**Affiliations:** 1 Department of Biology, University of New Mexico, Albuquerque, NM 87131 United States; 2 Department of Integrative Biology, University of California, Berkeley, Berkeley, CA 94720 United States; 3 Department of Botany, University of British Columbia, Vancouver, BC V6T 1Z4 Canada; 4 Department of Evolution, Ecology, and Organismal Biology, The Ohio State University, Columbus, OH 43210 United States

**Keywords:** Plant evolution, Evolutionary ecology, Experimental evolution

## Abstract

Hybridization is a common phenomenon, yet its evolutionary outcomes remain debated. Here, we ask whether hybridization can speed adaptive evolution using resynthesized hybrids between two species of Texas sunflowers (*Helianthus annuus* and *H. debilis*) that form a natural hybrid in the wild (*H. annuus* ssp. *texanus*). We established separate control and hybrid populations and allowed them to evolve naturally in a field evolutionary experiment. In a final common-garden, we measured fitness and a suite of key traits for these lineages. We show that hybrid fitness evolved in just seven generations, with fitness of the hybrid lines exceeding that of the controls by 14% and 51% by the end of the experiment, though only the latter represents a significant increase. More traits evolved significantly in hybrids relative to controls, and hybrid evolution was faster for most traits. Some traits in both hybrid and control lineages evolved in an adaptive manner consistent with the direction of phenotypic selection. These findings show a causal pathway from hybridization to rapid adaptation and suggest an explanation for the frequently noted association between hybridization and adaptive radiation, range expansion, and invasion.

## Introduction

Although historically regarded as a transitory or rare phenomenon^1–3^, natural hybridization is currently recognized as common in plants (occurring globally in 40% of families^4^ and involving up to 25% of species in some floras)^5^, important in animals (frequency of interbreeding species: 0.1–3%)^6^ with up to 25% in some groups^5^, and increasingly found in fungi (reviewed in^7^). Arguing from theory, researchers have hypothesized for decades that hybridization can act as an evolutionary stimulus^8–13^. Evolution is constrained by the availability of standing genetic variation^14,15^. Novel genetic material can arise via two main mechanisms: either by new alleles from *de novo* mutations^16,17^ or via introgression of alleles from other populations or species (reviewed in^18–20^). In fact, hybridization has been shown to provide sources of new genetic material (reviewed in^8,21^, see examples in^22,23^) and there is mounting evidence in numerous systems that hybridization is associated with adaptation, speciation, and radiation^8–10,24^. For instance, in a meta-analysis, naturally-occurring hybrids had increased invasion potential (measured via proxies such as fecundity and size) relative to their progenitor nonhybrid species^25^. However, most of this evidence is correlational in nature, thus the causal nature of these relationships needs to be investigated empirically with field experiments.

Evolutionary experiments allow for the observation and characterization of evolutionary change in real time using baseline conditions that are known with a high degree of certainty. They can address many questions, including those related to adaptation, evolutionary tradeoffs, population genetic parameters, and other lineage-specific evolutionary hypotheses^26–28^. Conducting these experiments in a field setting allows for the assessment of changes under realistic conditions that include multidimensional selective pressures and relevant genotype-by-environment interactions^29^. Such studies (those that take place in the field, involve some type of manipulation, and last for multiple generations *in situ*) on the effects of hybridization have so far been carried out exclusively in crop-wild systems. These studies have documented that natural selection favors wild alleles and phenotypes in some *Helianthus* crop-wild hybrids^30^, and in *Raphanus*, crop-wild hybrids outperform nonhybrids in terms of survival and fecundity^31–34^. However, evolutionary outcomes of hybridization in crop systems may differ from those in wild systems, as environments are homogenized in the former^35^ and population genetic parameters and loci under selection differ between agricultural and wild settings^36^.

Here, we present a unique field experimental evolution study investigating whether natural hybridization can speed adaptation in the wild. We focus on the rate of adaptation (as well as the phenotypic endpoints) as this component of hybrid evolution has been little studied. As a model system, we used three taxa of annual Texas sunflowers, including the annual sunflower *Helianthus annuus* ssp. *annuus*, the hybrid-derived subspecies *H. annuus* ssp. *texanus*, and the cucumber-leaved sunflower *H. debilis* ssp. *cucumerifolius*. All taxa are annuals that are wild and native to North America: *H. a. annuus* is geographically widespread across nearly the entire continent, while *H. a. texanus* and *H. debilis* are both centered in Texas. The subspecies *H. a. texanus* is locally-adapted to the environmental conditions in Texas (see fitness comparisons in^37^) and has long been considered the product of natural introgression of Texas-adapted *H. debilis* alleles into the widespread species *H. a. annuus*^38,39^.

We used an eight-year field experiment to examine adaptive evolution in initial, multiple intermediate, and final generations of control (nonhybrid) and resynthesized hybrid (*H. debilis* × *H. a. annuus* backcrossed to *H. a. annuus*) populations in a common-garden setting. Examination of intermediate generations allows for fine-scale temporal resolution of evolutionary rates. We measure evolutionary changes in fitness and morphology, focusing on a suite of 27 ecophysiological, phenological, architectural, resistance/palatability, and herbivore damage traits to obtain a comprehensive picture of phenotypic evolution. We further use genomic data to detect allelic changes due to local gene flow. We ask: (1) does hybrid fitness evolve compared to controls? (2) do key traits evolve more rapidly in hybrids relative to controls?, and (3) can trait evolution be predicted by initial phenotypic distance from the locally-adapted phenotype?

## Results

### Hybrid fitness evolution in a field experiment

**Figure 1:**
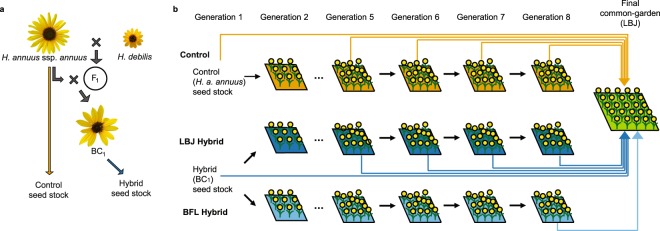
Field experimental evolution design. (**a**) Resynthesized hybrids were created by crossing *H. a. annuus* with *H. debilis* to form an F_1_ generation, then backcrossing this F_1_ generation to *H. a. annuus* to establish a hybrid BC_1_ seed stock. Sizes of illustrated inflorescences are approximately proportional to actual. (**b**) Initial populations of 500 individuals of Control (*H. a. annuus*, orange background) and Hybrid (BC_1_, dark blue background) were established in 2003 at separate plots at Lady Bird Johnson Wildflower Center (LBJ). A second Hybrid line was established at the Brackenridge Field Laboratory (BFL, light blue background). Lines were allowed to establish and reproduce naturally *in situ* for seven generations. Each generation, seeds were collected and stored from 96 randomly-chosen individuals per line. In 2017, a final common-garden was planted at LBJ with both control and hybrid seeds from generations one and five through eight for LBJ, generations one and eight for BFL, and accessions of locally-adapted *H. a. texanus* (not shown) for comparison.

**Figure 2:**
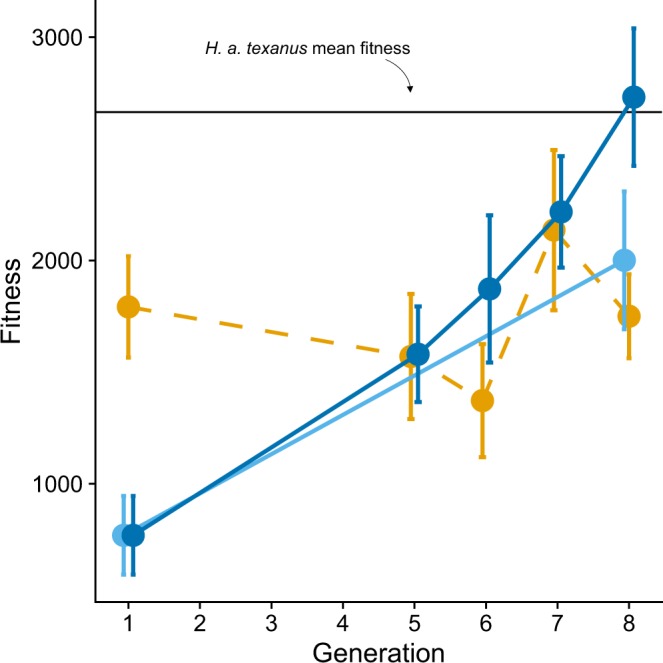
Hybrid fitness evolves through time. Mean fitness values (seed production) +/− SEM for control (orange) and hybrid (blue) lines are shown. Hybrid fitness evolved at both LBJ (dark blue) and BFL (light blue) (95% credible intervals for modeled slope in Bayesian analysis are positive and do not overlap zero). Control fitness did not change (95% credible interval overlaps zero). The solid black line is the locally adapted wild hybrid (*H. a. texanus*) mean fitness value for comparison.

We synthesized a hybrid population by creating an F_1_ hybrid (*H. a. annuus* × *H. debilis*) and back-crossing it to *H. a. annuus*, resulting in BC_1_ individuals with approximately 75% *H. a. annuus* and 25% *H. debilis* genetic backgrounds to mimic the hypothesized genetic composition of the ancestors of the natural hybrid lineage^38^ (Fig. 1a). We established separate plots with 500 sunflower individuals in two locations approximately 14.5 km apart in central Texas in 2003, Lady Bird Johnson Wildflower Center (LBJ) and the Brackenridge Field Laboratory (BFL) (see Methods for details). At LBJ, we established both hybrid (BC_1_) and control (*H. a. annuus*) lines (separated by 260 m), but due to space limitations only a hybrid line was established at BFL. For all experimental lineages, the initial allelic composition was derived from sources hundreds of kilometers distant from the study site (see Methods and Supplementary Fig. 1), and indeed neither hybrid  nor control populations were locally-adapted relative to local *H. a. texanus* (see generation 1 fitness in Fig. 2a). Thus, the design simulates a colonization event of a novel region, coupled (or not) with a hybridization event. Wild *Helianthus* neighbors were removed every spring to limit movement of alleles into or between the experimental plots (see Methods for details). This helped to reduce, but not eliminate, gene flow from local sources (see Supplementary Methods, Supplementary Fig. 2). Populations were allowed to reproduce naturally through generation 8; each generation we collected leaves from 96 individuals for genetic analyses and stored achenes (referred to as seeds hereafter) for common garden trials. In 2017, we established a final common garden at LBJ with multiple generations of controls and LBJ hybrids, the final generation of the BFL hybrid lineage, and multiple wild *H. a. texanus* accessions for comparison (Fig. 1b).

We tested our fundamental hypothesis, that hybridization can increase the rate of adaptation, by comparing the rate of fitness change across seven generations between hybrid and control populations. We estimated fitness as the total seed output of each individual (see Methods for details). We built Bayesian linear models in JAGS (Plummer 2003) to regress standardized fitness on generation and interpret significant positive slopes (β) as evolution of increased fitness. Control fitness did not change across generations (slope = 0.001, 95% credible interval = [−0.047, 0.047], Fig. 2). Hybrid fitness significantly increased through generational time (for LBJ hybrids: slope = 0.154, 95% credible interval = [0.096, 0.211], for BFL hybrids: slope = 0.115, 95% credible interval = [0.052, 0.180], Fig. 2). See Supplementary Table 1 for raw fitness values. Using a conservative Bayesian approach, we found that at LBJ control fitness exceeded hybrid fitness at generation 1 (mean standardized difference = −0.289, significant at 80% credible level [−0.535, −0.047], Fig. 2). Control and hybrid fitness values did not significantly differ in generations 5–7 at LBJ, but hybrid fitness exceeded control fitness in generation eight (by 56%; mean standardized difference = 0.320, significant at 95% credible level [0.007, 0.656], Fig. 2, Supplementary Table 2). Generation eight BFL hybrids had slightly higher fitness (14%) than LBJ controls, but this was not a significant difference (mean = 0.099, 95% credible interval = [−0.179, 0.429]. Thus, while both hybrid lineages were able to overcome low fitness levels in the early-generation (common in hybrids^40,41^ see meta-analysis in^25^) and evolve significantly, they significantly exceeded control fitness in one of the cases. The larger increase in performance of LBJ vs. BFL hybrids over controls may reflect local adaptation in the former to the common garden site, since the final common-garden was planted at home site for LBJ control and hybrid lines but was 14.5 km distant from the home site of the BFL hybrids.

### More traits evolved significantly in hybrids than in controls

**Figure 3:**
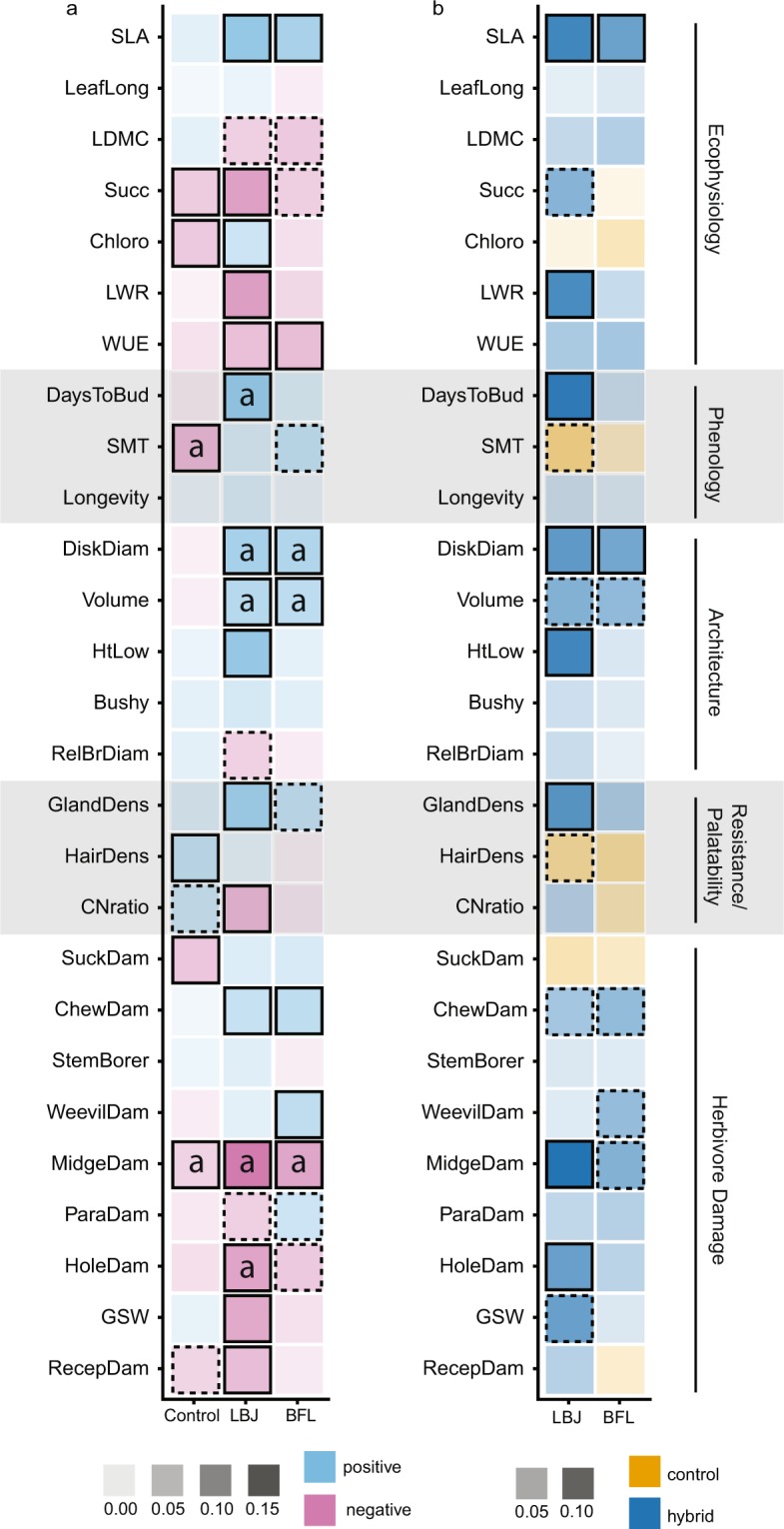
Hybridization leads to changes in the direction and magnitude of trait evolution. (**a**) Heatmaps for trait value changes through time for each of 27 different traits in controls and hybrids (LBJ and BFL). Color indicates whether the trait values increased (blue) or decreased (pink) through time. Squares with an “a” (“adaptive”) indicate that both selection gradients (measured in generation 1) and evolution through time were statistically significant and in the same direction, evidence that these traits evolved adaptively. Shading intensity increases with the absolute value of the posterior estimate. (**b**) Heatmaps measuring the degree to which traits in control and hybrid populations evolved at different rates, with either the absolute value of control (orange) or hybrid (blue) trait change estimates being steeper, and shading intensity increasing with a greater difference in steepness. Across both panels, squares outlined in black were significant at the 95% credible level, those outlined with black dashed lines were significant at the 80% credible level, and those with no outline were not significant.

We tracked evolution of ecophysiological, phenological, architectural, resistance/palatability, and herbivore damage traits (see Table 1, Methods). To determine which traits evolved through time, we ran the same Bayesian linear regression models as we did for fitness separately for each of the 27 traits. In the control population, six traits out of 27 evolved with strong support (significant at the 95% credible level), while two additional traits evolved with moderate support (significant at the 80% credible level) (Fig. 3a). In the LBJ hybrid population, 16 traits out of 27 evolved with strong support and an additional three traits evolved with moderate support, while in the BFL hybrid population seven traits evolved with strong support and an additional 6 traits evolved with moderate support (Fig. 3a). The number of traits evolving in controls and LBJ hybrids differed significantly for traits that evolved with strong support (Χ^2^ = 4.55, df = 1, p = 0.033) and for those with both strong and moderate support (Χ^2^ = 4.48, df = 1, p = 0.034). The number of traits evolving in controls and BFL hybrids did not differ significantly for traits evolving with strong support (Χ^2^ = 0.07, df = 1, p = 0.782) or traits evolving with both strong and moderate support (Χ^2^ = 1.19, df = 1, p = 0.275). See Supplementary Table 1 for raw trait data summaries and Supplementary Table 3 for full results from the Bayesian regression analyses.

**Table 1:** Traits measured in this study along with abbreviations and units, and trait evolutionary rates for control and hybrid populations, measured in haldanes (proportional change over generational time elapsed).

Trait	Abbreviation	Unit	Evolutionary Rates (haldanes)		
			Control	Hybrid (LBJ)	Hybrid (BFL)
Fitness	Fitness	Viable achenes/plant	−0.002	0.012	0.006
***Ecophysiological traits***					
Specific leaf area	SLA	cm^2^ ∙ g^−1^	0.004	0.020	0.018
Leaf longevity	LeafLong	days	0.000	0.002	−0.002
Leaf dry matter content	LDMC		0.000	−0.007	−0.010
Leaf succulence	Succ		−0.006	−0.013	−0.008
Leaf chlorophyll content	Chloro	SPAD reading	−0.008	0.005	−0.004
Leaf length:width ratio	LWR		−0.002	−0.016	−0.007
Water-use efficiency	WUE	δ 13C	−0.004	−0.011	−0.012
***Phenological traits***					
Bud initiation time	DaysToBud	days	0.001	0.028	0.005
Seed maturation time	SMT	days	−0.012	0.006	0.010
Plant longevity	Longevity	days	0.000	0.006	0.002
***Architectural traits***					
Disk diameter	DiskDiam	mm	0.000	0.013	0.012
Plant volume	Volume	cm^3^	−0.001	0.014	0.010
Height of lowest branch	HtLow	cm	0.003	0.018	0.001
Bushiness	Bushy		0.004	0.005	0.003
Relative branch diameter	RelBrDiam		0.005	−0.006	−0.001
***Resistance/palatability traits***					
Glandular trichome density	GlandDens	mm^−2^	0.002	0.011	0.008
Nonglandular trichome density	HairDens	mm^−2^	0.008	0.001	0.001
Leaf Carbon:Nitrogen	CNratio		0.008	−0.013	−0.002
***Herbivore damage traits***					
Leaf-vascular-tissue damage	SuckDam	%	−0.004	0.005	0.006
Leaf-chewing damage	ChewDam	%	0.001	0.012	0.013
Stemborer damage	StemBorer	Holes per plant	0.002	0.003	−0.001
Weevil damage	WeevilDam	Petioles per plant *(Rhodobaenus* sp.)	−0.002	0.003	0.012
Midge damage	MidgeDam	Fraction seeds killed (*Neolasioptera helianthis*)	−0.005	−0.023	−0.017
Parasitoid damage	ParaDam	Fraction *N. helianthis* parasitized	−0.002	−0.011	0.008
Hole damage	HoleDam	Fraction seeds damaged by holes (*Isophrictis* sp.)	−0.001	−0.016	−0.009
Gray seed weevil damage	GSW	Fraction seeds killed (*Smicronyx sordidus*)	0.004	−0.008	−0.003
Receptacle damage	RecepDam	Average number of receptacle holes *(Isophrictis* sp.)	−0.003	−0.012	−0.001
***Global average***		(Absolute value, across all traits)	0.003	0.011	0.007

### Some traits evolved adaptively

Although traits evolved in both controls and hybrids, not all evolution was necessarily adaptive in nature, as it could have been driven by demographic factors such as genetic drift or by genetic correlations with other traits under selection; we note that many traits were correlated (Supplementary Fig. 3), but the strength of correlations did not differ between controls and hybrids. To assess which instances of trait evolution were adaptive, we compared the direction of trait changes with a phenotypic selection analysis (PSA) performed in 2003, the initial year of the experiment^37,42^. If both the evolution of the trait and the selection gradient (β, the partial regression coefficients from a multiple regression, see Methods) were significant and in the same direction, we interpreted this as evidence for adaptive evolution (see Supplementary Table 4 for PSA results). In controls, two out of six traits with significant selection gradients fit these criteria for adaptive evolution, while in LBJ and BFL hybrids, five out of ten and three out of six traits with significant selection gradients fit these criteria (Fig. 3a), though this difference in proportions between treatments is not significant (LBJ versus controls: Χ^2^ = 0.033, df = 1, p = 0.855, BFL versus controls: Χ^2^ = 0.033, df = 1, p = 0.855). Conversely, we examined how many of the traits that evolved significantly also evolved adaptively. In controls two out of five traits that evolved did so in an adaptive manner, while four out of 16 did so for LBJ hybrids and three out of six did so for BFL hybrids (Fig. 3a). These differences in proportion were not significant (LBJ versus controls Χ^2^ = 0.025, df = 1, p = 0.874, BFL versus controls Χ^2^ = 0.011, df = 1, p = 0.916). Note that not all traits measured in 2017 were included in the 2003 phenotypic selection analysis.

### Rates of trait evolution are faster in hybrids relative to controls

We resampled estimates from the posterior distributions of the Bayesian slope estimates of trait change through time to create new posterior distributions comparing trait evolution in hybrids versus controls, subtracting the absolute value of control slope estimates from the absolute value of hybrid slope estimates, a conservative approach^43^. For eight traits the LBJ hybrids had steeper slope values than controls (with strong support) and four additional traits showed moderate support for a steeper slope (Fig. 3b). For two traits the BFL hybrids had steeper slope values than controls (with strong support) and four additional traits showed moderate support for a steeper slope Fig. 3b). There were zero traits for which the controls had strong support for steeper slopes, though two traits had moderate support (LBJ, Fig. 3b). Differences in the number of traits evolving faster in LBJ hybrids versus controls were significant when examining those with strong support (Χ^2^ = 8, df = 1, p = 0.005) and those with both strong and moderate support (Χ^2^ = 7.14, df = 1, p = 0.007). Differences in trait evolution for the BFL hybrids versus controls were not significant when examining those with strong support (Χ^2^ = 2.00, df = 1, p = 0.157) or those with both strong and moderate support (Χ^2^ = 2.00, df = 1, p = 0.157). As an additional metric, we measured the rate of trait evolution in haldanes (proportional change over generational time elapsed)^44^. Control traits evolved faster than LBJ hybrids in only three traits (mean absolute rate = 0.003 haldanes, range 0.000–0.012), while the LBJ hybrids evolved faster in the remaining 24 traits (mean absolute rate = 0.011 haldanes, range = 0.001–0.028), a significant difference (Χ^2^ = 16.3, df = 1, p < 0.001). Controls evolved faster than BFL hybrids in ten traits, while the BFL hybrids evolved faster in the remaining 17 traits (mean absolute rate = 0.007 haldanes, range 0.001–0.018), again a significant difference (Χ^2^ = 9.80, df = 1, p = 0.002) (Table 1).

### Distance from the locally-adapted phenotype predicts trait evolution

**Figure 4:**
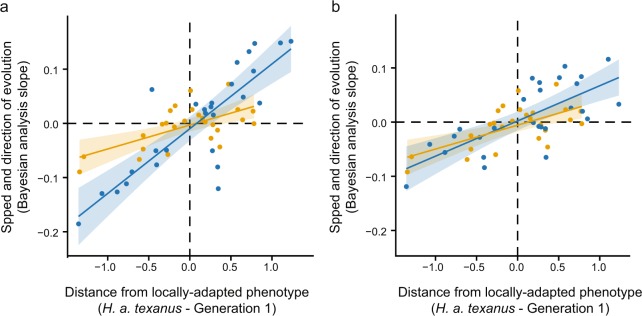
Distance from the locally-adapted phenotype predicts trait evolution. For both control (orange) and hybrid (blue) lineages, the rates of evolution for individual traits (using the slope values from the Bayesian evolution analyses) are significantly correlated with the distance from the initial mean trait value (BC_1_ and initial *H. a. annuus* generation for hybrids and controls, respectively) to the mean trait value of *H. a. texanus*. Each point represents an individual trait. Note that the same control lineage originally grown at LBJ (orange) is shown in both figure panels. (**a**) LBJ hybrid lineage vs. control. (**b**) BFL hybrid lineage vs. control.

Traits evolved in both hybrids and controls at different rates (above). We asked whether the initital phenotypic distance to the locally-adapted taxon (*H. a. texanus*) could predict the rate and direction of trait evolution. To do so, we computed the distance from the standardized average trait value of our experimental plants in generation 1 (hybrids and controls separately) to the average trait value of *H. a. texanus* planted in the garden. We used a simple linear regression to relate distance to the slopes from the Bayesian trait evolution analyses. Control and hybrid (both LBJ and BFL) evolutionary rates were both positively and significantly correlated with distance from the locally-adapted phenotype, meaning that traits with initial values far from those of the local phenotype tended to evolve faster than traits starting with trait values similar to the local phenotype, and in a direction toward the values of *H. a. texanus* (Fig. 4). However, the control estimate (coefficient = 0.053, p = 0.001) was not as strong as the LBJ hybrid estimate (coefficient = 0.120, p < 0.001), but similar to the BFL hybrid estimate (coefficient = 0.065, p < 0.001). This pattern indicates that the LBJ hybrid population was able to approach a locally-adapted phenotype more rapidly than controls, even when hybrids and controls started at an equal level of presumed maladaptation (i.e., for a given initial phenotypic distance between the experimental populations and *H. a. texanus*, the estimated rate of evolution is faster for hybrids than controls, Fig. 4a). See Supplementary Table 5 for values used.

### Hybrid populations were susceptible to local gene flow

We found outside alleles in most, but not all hybrid samples (note that outside alleles could not be surveyed in the control population, see Methods). Although the proportion of outside alleles varied between individuals, a fraction of hybrid samples (14 LBJ and 17 BFL) had virtually no outside alleles (<0.5%), suggesting these samples had no outside ancestry and supporting the validity of the test statistic. For the LBJ experimental hybrid population, the percentage of admixed individuals increased with generation, from 0% in generation 1 (the BC_1_ generation), to 78% in generation 3 and 100% by generation 6 (Supplementary Fig. 2a). The BFL hybrid population had a similar trajectory, from 83% in generation 3 to 100% in generation 7 (Supplementary Fig. 2). The average percentage of novel alleles increased from generation 3 to generation 5, but subsequently stabilized at ~5%, suggesting fewer migrants in later generations (Supplementary Fig. 2b,c).

## Discussion

Hybridization can result in adaptive introgression of fitness-enhancing alleles from one species into another^45^. While *de novo* mutations are biased toward deleterious effects and occur at relatively low rates over time, introgressing alleles can be introduced simultaneously in large numbers. Further, introgressing alleles have been tested by natural selection in the donor species, albeit in a different genetic background, and thus may be more likely to be beneficial. This mechanism is one potential explanation for the observed rapid increase in fitness and evolution of key traits in hybrids observed in our experiment. Adaptive introgression has been proposed in many examples (reviewed in^18,19,46^), including this system. Heiser originally proposed that *H. a. texanus* is the product of natural hybridization between *H. debilis* and *H. a. annuus*, with incorporation of genetic material from *H. debilis* allowing for the southward range expansion of *H. annuus* into Texas.

Previous work in this system found evidence for adaptive introgression of traits related to abiotic tolerance^42^ and herbivore resistance^37^ and identified putative QTL underlying these traits^47^. Our experiment finds rapid evolution in both traits and fitness in resynthesized hybrids, potentially due to the introgression of traits and alleles from *H. debilis*. Traits in all broad categories (ecophysiology, phenology, architecture, resistance/palatability, and herbivore damage) tended to evolve faster in hybrids than in controls (Fig. 3). The potential adaptive nature of these traits has been addressed in more detail in previous work (see^37,42^), but briefly we note that the damage done by seed predators (seeds killed by midges, MidgeDam; seeds damaged by *Isophrictis* holes, HoleDam; and seeds killed by seed weevils, GSW) is a strong selective pressure, apparently resulting here in decreased rates of damage in later generations. Other changes in ecophysiology or architecture may be associated with adaptation to the local abiotic environment, although we did not test for specific causation here. For instance, in both hybrid lines, specific leaf area (SLA) evolved higher values, perhaps in response to the warmer conditions in the study area relative to those experienced by the more northern *H. a. annuus* parent. Additionally, phenotypic distance from the locally-adapted phenotype (*H. a. texanus*) is correlated with the speed of evolution, indicating that this experiment may be “replaying” the natural history of hybridization, introgression, and adaptation in this system.

A competing (but not mutually-exclusive) explanation for the observed rapid evolution in hybrids is that, relative to the control genome, the hybrid genome may have permitted more rapid introgression of local alleles from wild *H. a. texanus* in the local environment. While we reduced local gene flow by removing wild *Helianthus* individuals near our experimental plots, we did not eliminate it, and novel alleles began to accumulate in the hybrid populations, stabilizing at around 5% of loci by generation 6 at both LBJ and BFL (Supplementary Fig. 2). The genomes of the original BC_1_ hybrid plants, and plants of subsequent generations, may have been more porous than those of controls due to a low diversity of self-incompatibility (SI) alleles, as has been found in *Senecio squalidus* in the British Isles^48^. In this scenario, a large fraction of the incoming pollen from local sunflowers would have had unique SI alleles relative to experimental hybrids, and thus would have had fertilization advantage. An alternative mechanism for increased porosity posits that hybrids may have had relatively high genetic load (relative to controls) via outbreeding depression (as evidenced by low initial fitness of the hybrids, Fig. 2), which in turn would lead to higher than neutral introgression of local alleles^49,50^. Under this mechanism, offspring sired by local pollen would have had higher fitness, resulting in a reduction in genetic load and an increased frequency of local alleles in subsequent generations. Rather than an experimental artifact, this porosity is likely a general a feature of natural hybridization events, where initial populations of interspecific hybrids are small in size and are likely to have one or both of the above-described genomic features (low SI allele diversity, high genetic load). We posit that historical formation of the wild hybrid *H. a. texanus* may have been shaped by this process as well, with high porosity and allele-sharing between multiple, independently formed early-generation hybrid populations contributing to rapid spread of advantageous alleles.

We found higher rates of trait evolution in hybrids relative to controls, both in terms of the slope of change over time (Fig. 3b) and in evolutionary rates measured in haldanes (Table 1). The latter rates allow comparisons with previous studies in plants over similar generational time periods. Bone and Farres^51^ compiled microevolutionary rates in plants across 23 studies, 15 of which include estimates in haldanes. Rates ranged from 0–0.808, with a mean of 0.158 haldanes. Most rates fell at the lower end of the distribution (<0.05 haldanes) with a median of 0.047 haldanes and only four studies with rates higher than 0.5 haldanes^51^. The rates we estimated in our study (means of 0.0003, 0.011, and 0.007 haldanes for controls, LBJ hybrids, and BFL hybrids, respectively, range 0 to 0.028), are well within this wide range.

Here we show that, despite initial low fitness values, *Helianthus* hybrids rapidly evolved higher fitness than non-hybrid controls. Further, key traits associated with ecophysiology (such as specific leaf area, leaf succulence, leaf length-width ratio), phenology (bud initiation time, seed maturation time), architecture (floral disk diameter, branching height), and herbivore resistance (seeds killed by midges or holes) evolved faster in hybrids relative to controls, perhaps enabling hybrids to acquire resources and produce viable offspring more effectively. Our results provide experimental support for the long-hypothesized connection between hybridization and more rapid adaptive evolution and provide a set of potential mechanisms for the long-noted association of hybridization with diverse evolutionary phenomena including adaptive radiations, invasions, and range expansions. However, an important caveat is that we have been able to examine only one control and two hybrid populations, given logistical constraints inherent in conducting a large field experiment over eight years. Replication in studying the evolutionary outcomes of hybridization will need to be built up across (rather than within) studies, as has been done for other evolutionary questions requiring intensive field-based investigation. For instance, a series of experiments transplanted populations of the Trinidadian guppy *Poecilia reticulata* between environments, there by manipulating exposure to predators^52–55^. Each study was conducted using either microcosms or field systems in different rivers or tributaries, building on each other and on previous observational work to form a more complete picture of life history and color pattern evolution in response to predators (reviewed in^28^). Replication of field experiments within our *Helianthus* system and other plant and animal systems should provide additional evidence on the degree to which hybridization can speed adaptive evolution, while elucidating details regarding the context-dependent nature of hybrid evolution.

## Methods

### Establishment of hybrid and control lineages

Our design simulated the arrival of a non-locally-adapted taxon (*H. a. annuus*) to the study area from the north, followed either by hybridization with the local *H. debilis* (BC_1_ hybrid lines) or not (*H. a. annuus* control line). These populations then evolve in, and exchange genes with, a matrix of other hybrid populations, similar to the expected scenario during the historical formation of *H. a. texanus*. Note that the parental species *H. debilis* is native to the study area, and thus would not be an informative non-hybrid control for the rate of local adaptation, since it is presumably already locally adapted. The BC_1_ generation was obtained by first mating *H. debilis* from Texas to wild *H. a. annuus* from Oklahoma to produce F_1_ progeny in the greenhouse (Supplementary Table 6, see details in^37^). To produce enough BC_1_ seed for replicate field populations, a single progeny from the F_1_ generation was selected and propagated vegetatively to produce 14 F_1_ clones. A single *H. a. annuus* pollen donor from north Texas (the recurrent parent) was then mated to the F_1_ clones. Controls were field-collected *H. a. annuus* from the recurrent parent population “RAR59” (see Table 1 in^37^), consisting of roughly 10 seeds from each of 50 maternal sibships.

Seeds for both hybrids and controls were germinated, planted in field soil in peat pots, and grown in the greenhouse for one month before transplanting. One pair of control (*H. a. annuus*) and hybrid (BC_1_) populations were established at Lady Bird Johnson Wildflower Center (LBJ, 30.184°N, −97.877°W), with plots separated 260 meters and a dense copse of trees to limit gene flow between plots. To assess the generality of hybrid responses in different parts of the *H. a. texanus* range, a second hybrid population was established approximately 14.5 km away at the Brackenridge Field Laboratory (BFL, 30.282°N, −97.780°W). Space limitations prevented the establishment of a control population at BFL. All three populations were initiated with 500 individuals in late March 2003. The source populations for the BC_1_ line are far away from LBJ (*H. debilis* F_1_ parent: ~300 km; *H. a. annuus* F_1_ parent: ~650 km; *H. a. annuus* recurrent parent: ~375 km; see Supplementary Fig. 1, Supplementary Table 6), as is the source population for the control line (~375 km), so both types of experimental populations should be less locally adapted to the establishment sites than is either *H. a. texanus* or the parental species *H. debilis*. Populations were allowed to evolve naturally without human interference, with two exceptions. First, as annual sunflowers are early-successional species and require annual soil disturbance to maintain population sizes, we disturbed each plot each winter using a rototiller. Second, to provide some isolation to the experimental populations, we reduced (but did not eliminate, see Supplementary Fig. 2) the rate of local gene flow, by pulling all wild sunflowers that emerged within a 250 m buffer surrounding each plot prior to flowering each year.

Each year, we collected seeds and leaves from 96 individuals per population for use in the final common-garden and for genetic analyses, respectively. Seeds were stored at 20 °C in paper coin envelopes in sealed plastic tubs with drierite (W. A. Hammond DRIERITE Co., Ohio, USA) to maintain low humidity. The experiment had to be terminated after eight generations because of a change in land use at the host site.

### Final common-garden

Stored seeds were germinated at the University of New Mexico (UNM). These included seeds from hybrid and control lineages, as well as seeds from wild *H. a. texanus* (see Supplementary Table 6 for source population information). Germination protocols are described in detail in^37^ and included hand-scarification of seeds germination on filter paper, and transplantation into peat pellets (Jiffy J3675, Oslo, Norway), followed by roughly one month of growth in the UNM greenhouses. Over ten thousand seeds were scarified for this study, with an average germination rate (across all lines) of 36%. The final common garden was planted at LBJ between April 2 and April 4, 2017. One-month old seedlings were transported to Texas and planted 90 cm apart in rows 1 m apart. We aimed to plant 60 individuals per lineage for final generation and wild *H. a. texanus* lines and 30 per lineage intermediate generations, using approximately equal numbers of seeds per maternal line to avoid overrepresenting any individual half-sibling families. Due to variable germination rates, the actual number of individuals planted varied (Supplementary Table 7). In particular, low seed availability and low germination rates (the latter likely due to age-associated mortality in storage, presumably associated with seed pathogens acquired from the field) meant that we were unable to include generations two through four in the final common garden. Note that in contrast, generation one plants were formed in the greenhouse, and therefore this generation was not exposed to these putative seed pathogens. The garden included 1002 plants total, 615 used in this study. Heavy rainfall just prior to planting meant that plants were able to successfully establish without planned hand-watering.

### Trait measurements

Traits were measured as in^37,42^; see Table 1 for traits, abbreviations, and units. Briefly:

### Ecophysiological traits

Specific leaf area (SLA, cm^2^ ∙ g^−1^) is the ratio of leaf area to mass and a measure of overall leaf construction costs (higher values associated with less cost per light-absorbing area). Leaf succulence (Succ) is calculated as (leaf wet mass − dry mass)/wet mass, while leaf dry matter content (LDMC) is calculated as leaf dry mass/wet mass. Leaf length to width ratio (LWR) is an estimate of the narrowness of the leaf and is calculated as leaf length/leaf width. Leaf water use efficiency (WUE) is the rate of carbon gained via photosynthesis per unit of water lost via transpiration, measured using δ^13^C. Leaf longevity (LeafLong) is the length of time that a leaf remains on the plant, measured in days. Two fully expanded leaves per plant were selected in the period before first flowering, one for WUE and one for the remaining leaf traits. An expanding leaf of approximately 4 cm was selected and tagged with a jewelry tag to track leaf longevity. Chlorophyll content (Chloro) was estimated using a SPAD meter (Spectrum Technologies, Aurora, IL), averaged across five measurements. Wet mass was measured on a microbalance, leaves were scanned using a flatbed scanner, and leaves were dried in a drying oven until constant mass was reached, and dry mass was then measured. One ca. 3 mg sample from dried leaf disk tissue (taken using a #7 cork borer) per plant was weighed in tin and analyzed for Carbon, Nitrogen, ^13^C, and ^15^N content at the University of New Mexico Center for Stable Isotopes. To reduce costs, only a subset of individuals was submitted for isotope analysis and leaf Carbon:Nitrogen ratio (CNratio) (Supplementary Table 7).

### Phenology

Phenological status of all plants in the final common garden was assessed every third day beginning on May 1 until the majority of plants had senesced in early November. From these assessments, we calculated bud initiation time (DaysToBud) as the number of days between transplanting and the first appearance of the immature apical flowering head, seed maturation time (SMT) as the number of days between the end of stigma receptivity and achene maturity for the apical head, and plant longevity (Longevity) as the number of days between transplanting and mortality. For a small number of plants remaining alive after the last census date in November, we added one or two weeks to longevity based on the plant’s appearance (10 and 3 individuals, respectively). Eliminating these outliers from the analyses did not affect results.

### Architectural traits

Disk diameter (DiskDiam, mm) is the diameter of the central disk of the apical flowering head measured during stigma receptivity. Height of the lowest branch (HtLow, cm) is the height of the lowest branching point on the primary stem. Bushiness (Bushy, a measure of higher-order branching) was estimated as the mean branch position of all flowering heads on the plant^56^, where heads originating on the main stem have a branch position of 1, heads from a primary branch have a branch position of 2, and heads from a secondary branch have a position of 3. Relative branch diameter (RelBrDiam) is a measure of investment in branches relative to the main stem and is estimated as the average branch diameter across all primary branches with diameters at the base > = 3 mm, divided by the stem diameter. Plant volume (Volume, cm^3^) is an estimate of overall plant size and is calculated using the equation for the volume of a cylinder, *V* = π × *r*^2^ × *l*, where *r* is half the diameter of the primary stem and *l* is the height of the plant.

### Seed damage and fitness

For seed count and seed predation measurements, individual seed heads were enclosed with bags made of plastic mesh (DelStar Technologies, Delaware) after pollination but before seed release and collected throughout the season, with a goal of >4 heads per plant. At plant senescence, all remaining heads (both bagged and unbagged) were collected and counted. Damage to receptacles (the structures subtending the inflorescence) by head-feeding Lepidoptera was measured by counting the number of larval holes in a sample of one to eight mature receptacles per plant and taking the average (RecepDam). Seed damage was visually assessed under a dissecting microscope (Leica, Wetzlar, Germany). Seeds were sorted into categories including viable, parasitoid attacked (ParaDam), midge damaged (MidgeDam, the sunflower seed midge *Neolasioptera helianthis*; Diptera: Cecidomyiidae combined with parasitoid damage), hole damaged (HoleDam, *Isophrictis* sp.; Lepidoptera: Gelechiidae), and gray seed weevil damaged (GSW, *Smicronyx sordidus*; Coleoptera: Curculionidae). In total, 310,378 seeds were scored for this study (mean = 536 seeds per plant). Damage scores were calculated as fractions (number of seeds in each category/total seeds scored per plant). Viable seed production was chosen as the measure of fitness in these annual plants. Seed production was estimated by multiplying the total number of heads produced (bagged + unbagged) by the average number of viable seeds per bagged head.

### Resistance/palatability traits

Densities of glandular (GlandDens) and nonglandular (HairDens) trichomes were measured on a single dried leaf disk per plant. Trichomes were counted using a Leica MZ 125 compound light microscope (Leica, Wetzlar, Germany) under 5x magnification with a 1 cm × 1 cm reticle and converted to densities (measuring a 0.2 cm × 0.2 cm area, 0.04 cm^2^). The ratio of leaf carbon to nitrogen (CNratio) was estimated using values from the Center for Stable Isotopes (see above).

### Herbivore damage traits

Insect damage to leaves was scored twice for each plant, once in mid-June and once in late-July. Briefly, we scored percent cover of damage on three of the oldest leaves per plant caused by different types of herbivores and calculated a damage index *D* for each measured as percentage of leaf area, see^37^ for details. Composite damage indices for leaf-vascular-tissue feeders (SuckDam: Hemiptera, Homoptera) and for leaf chewers (ChewDam: Orthoptera, Lepidoptera, Diptera) were constructed by summing *D* scores for each of the component taxa. Stem and petiole damage were assessed in mid-June as a continuous trait, counting the number of lesions on all stems and petioles caused by *Rhodobaenus* weevils (Coleoptera: Curculionidae) (WeevilDam), and number of holes per plant caused by stem-boring larvae (StemBorer, Coleoptera, Lepidoptera).

### Statistical analysis: general

All analyses were carried out in R v3.3.1^57^. Prior to analysis, of the 615 original plants, we filtered out early transplant deaths (n = 9), plants apparently damaged during seed scarification that never developed a root system (n = 10), and plants that lived less than 75 days because the majority of traits could not be measured on these plants (n = 35; note that including these plants in analyses did not affect fitness results). Additionally, when analyzing architectural traits, we excluded plants with weevil damage to the primary bud, as these resulted in abnormal growth architectures. See Supplementary Table 7 for final counts of each line analyzed. Trait values across control and hybrid lines were standardized to a mean of zero and standard deviation of one.

### Trait evolution

We used Bayesian linear regression models to estimate the evolutionary change in phenotypic values of traits through time. We ran separate models for each trait (response variable, Eq. (1)), with generation as covariate, using separate slopes and intercepts for control and hybrid lines, where ${y}_{i}$ is the standardized value of each trait for individual $i$, ${x}_{i}$ is the covariate generation:

1 $${y}_{i}={\beta }_{0j}+{\beta }_{j}{x}_{i}+{{\boldsymbol{\epsilon }}}_{i}$$

*β*_0*j*_ is the intercept term for treatment *j* (control or hybrid) and *β*_*j*_ is the regression coefficient for treatment *j*. For the slope and intercept terms, we used normal priors with a mean of zero and variance of one, while the error term was assigned a gamma prior with shape and scale both equal to one. We implemented the models in JAGS v 4.3.0^58^ and report results from five MCMC (Markov Chain Monte Carlo) chains run for 100,000 iterations (with a burnin of the first 25,000 discarded), thinned every 25^th^ iteration, with 10,000 iterations saved for the posterior sample. We used traceplots to check for convergence, and verified that R_hat_ values were under 1.01 for all parameters^59^. Regression coefficients were deemed significant if the 95% credible intervals did not overlap zero. We used all 10,000 posterior samples to create posterior distributions for the difference between treatments by subtracting the values for the hybrid coefficient samples from the control coefficient samples and taking the absolute values of these differences. From these new distributions, we estimated means and 95% credible intervals, where intervals that did not overlap zero indicate that coefficients differ between treatments.

To explicitly compare fitness values for controls against hybrids for each generation (LBJ) or the final generation (BFL), we ran Bayesian models using the stan_glmer() function in the R library *rstanarm*^60^ using standardized fitness values as a response with a random terms for lineage. We increased adapt_delta to 0.999 to avoid divergent transitions and used N(0,100) priors. We ran each model using four chains for 2000 iterations each including a 1000 iteration burnin and checked R_hat_ values (<1.01) to ensure that the chains converged. We compared the posterior distributions by subtracting the samples of control fitness from the samples of the hybrid fitness, producing a mean and full distribution of the differences.

We calculated the allochronic evolutionary rates in haldanes, the proportional change over generational time elapsed (*H*, Eq. (2)), for both controls and hybrids using the method of ^44^:

2 $$H=\frac{\frac{{\bar{y}}_{8}-{\bar{y}}_{1}}{{s}_{p}}}{g}$$

where ${\bar{y}}_{8}$ is the mean natural log-transformed value of the trait in generation 8 and ${\bar{y}}_{1}$ is the mean natural log-transformed value of the trait in generation 1, *s*_*p*_ is the pooled standard deviation of the samples, and *g* is number of generations passed (*g* = 7).

### Phenotypic selection analysis

To determine whether evolution was adaptive for individual traits, we determined if the direction of trait evolution aligned with trait – fitness associations during 2003, the first field generation. We estimated selection differentials (*s*′) and selection gradients (β) from 2003 trait and fitness data separately for controls and hybrids at each home site (BFL and LBJ)^37,42^. Predictor variables were transformed to approach normality then standardized to a mean of 0 and standard deviation of 1 within each treatment. Linear selection differentials were estimated from the covariance of relative fitness within treatment (hybrid or control). Linear selection gradients were estimated from the partial regression coefficients from a multiple regression using the *lm*() function in R (see Supplementary Table 4 for traits used in the regression models). Note not all traits measured in 2017 were measured in 2003, e.g. Chloro, LWR, and ParaDam were not measured in 2003. Assumptions of normality were violated when using relative fitness values, so we generated 95% confidence intervals using bias-corrected bootstrap sampling of 10,000 replicates using the *boot*^61^ package in R. Phenotypic selection results for hybrids were published previously^37,42^, while results for controls are previously unpublished. In the current analyses, we included additional traits not included in previous analyses, so values of β (which depend on the phenotypic correlations of the focal trait with the other traits in the model, and with selection on those other traits) will necessarily differ somewhat from previously reported results. Full results are presented in Supplementary Table 4.

### Predicting the speed of evolution

For each trait, we calculated the distance from the mean value of the initial (2003) generation hybrids and controls to that of *H. a. texanus*, which we assume to have a locally-adapted phenotype. Across all traits, we estimated the correlation between this distance and the slope from the Bayesian analysis (as a measure of the speed of evolution) using the *lm*() function in R.

### Detection of local gene flow

We used genotyping-be-sequencing to determine if our experimental hybrid populations experienced local gene flow from outside the experiment (e.g., from naturally occurring *H. a. texanus* individuals) over the course of the study. We sequenced 90 samples from the original BC_1_ generation, plus 229 LBJ and 264 BFL samples from subsequent generations 3 to 8; material from generation 2 was not available. See Supplementary Methods for details on DNA extraction, library preparation, sequencing, and bioinformatics. Since our hybrid population is entirely derived from a single backcross, all variants should be found in the BC_1_ population at approximately 25%, 50% or 75% frequency. We did not attempt to examine the control population in this way, as its much higher starting level of allelic diversity would have made identification of outside alleles difficult or impossible. In our hybrid populations, variants that only appear in subsequent generations are likely to be the result of outside gene flow. To quantify this gene flow, we filtered for sites sequenced in at least 20 BC_1_ samples and catalogued all alleles present. We then looked in all subsequent generation samples and calculated the percentage of called variants that were not in our catalogue from the BC_1_s. See Supplementary Methods for full explanation of genetic work.

## Supplementary information

Supplementary Information
