# Supplementary material for: Hybridization speeds adaptive evolution in an eight-year field experiment
Source: Sci Rep. 2019 May 1;9:6746. doi: 10.1038/s41598-019-43119-4 (PMC6494830; doi:10.1038/s41598-019-43119-4)
Supplement: Supplementary file 1 — Supplementary Information [file 41598_2019_43119_MOESM1_ESM.pdf]

Hybridization speeds adaptive evolution in an eight-year field experiment

Nora Mitchell

Gregory L. Owens

Stephen M. Hovick

Loren H. Rieseberg

Kenneth D. Whitney

Supplementary Information

Includes:

- Supplementary Methods
- Supplementary Figures 1, 2, 3
- Supplementary Tables 1, 2, 3, 4, 5, 6, 7

Supplementary Methods

We used genetic sequencing to determine if our experimental hybrid populations experienced local gene flow from outside the experiment (e.g., from naturally occurring *H. a. texanus* individuals) over the course of the study. We did not attempt to examine the control population in this way, as its much higher starting level of allelic diversity would have made identification of outside alleles difficult or impossible. For the hybrids, we sequenced 90 samples from the original BC1 generation, 229 LBJ and 264 BFL samples from subsequent generations 3 to 8; material from generation 2 was not available. Leaf samples were taken each generation from adult plants in the field, with the exception that samples here designated as representing generations "3" and LBJ "8" were from greenhouse-grown plants germinated from seeds collected from the field the previous generation (that is, seeds that were collected from generations 2 and 7).

DNA was extracted from 40mg samples of fresh leaf tissue using Qiagen DNeasy 96 plant kits in conjunction with a Qiagen Mixer Mill MM 300 (Qiagen, Maryland, USA). Samples were sequenced using a two enzyme Genotyping-By-Sequencing protocol[1]. Briefly, 100 ng of genomic DNA was digested with the endonuclease PstI-HF and MspI. Fragments were ligated to adaptors including the Illumina sequencing adaptor and size selection was performed to retain amplified fragments of length between 300-500bp. To reduce the representation of repetitive sequences in the libraries, we performed a depletion step by treating the enriched libraries with Duplex-Specific Nuclease. Pair-end sequencing of 125 bp was performed on an Illumina HiSeq 4000, with 196 samples pooled per lane of sequencing (including samples not used in this study).

Reads from individual samples were demultiplexed using a custom perl script and trimmed for base quality and adapter sequences using Trimmomatic (v0.32)[2]. Reads were then

aligned to the *H. annuus* XRQ (v1.0) genome using NextGenMap (v0.5.0)3. Repetitive transposable elements represent a large fraction of the *H. annuus* genome and are challenging to call variants, so we removed all reads that entirely mapped to the annotated TE portions of the genome using samtools (v1.4)[4]. We then called variants using GATK (v3.7) using the HaplotypeCaller and GenotypeGVCF tools[5]. We subsequently filtered the resultant variant file to only include genotypes with >=5 reads, and sites with < 50 % missing data, > 3 minor allele count, 1 alternate allele and only SNPs (i.e. not indels or complex variants). Data from each experimental population was filtered separately and both contained the same BC1 samples, since both populations were derived from the same BC1 pool.

Since our hybrid population is entirely derived from a single backcross, all variants should be found in the BC1 population at approximately 25%, 50% or 75% frequency. Variants that only appear in subsequent generations are likely to be the result of outside gene flow. To quantify this gene flow, we filtered for sites sequenced in at least 20 BC1 samples and catalogued all alleles present. We then looked in all subsequent generation samples and calculated the percentage of called variants that were not in our catalogue from the BC1s. This represents the percentage of the genome that is novel by state. Note that gene flow may also introduce alleles already present in the founding population (that is, alleles that are novel by descent but not by state); such alleles are less likely to have an effect on the adaptive potential of the population, are difficult to identify, and are not considered further here.

For the LBJ experimental population, after filtering, we retained 118,410 biallelic SNPs. To include a site in our scan for local gene flow we required it to be genotyped in >= 20 BC1 samples, which resulted in 118,304 usable sites. Of these sites, our advanced generation samples were genotyped in 98,823 sites on average (54,965 to 105,461 sites). For BFL, we retained

125,783 biallelic SNPs and 125,720 usable sites. Our advance generation BFL samples were genotyped in 104,955 sites on average (75,457 to 112,932 sites).

References

1. Poland, J. A., Brown, P. J., Sorrells, M. E. & Jannink, J.-L. Development of high-density genetic maps for barley and wheat using a novel two-enzyme genotyping-by-sequencing approach. *PloS one* **7,** e32253 (2012).

2. Bolger, A. M., Lohse, M. & Usadel, B. Trimmomatic: a flexible trimmer for Illumina sequence data. *Bioinformatics* **30,** 2114–2120 (2014).

3. Sedlazeck, F. J., Rescheneder, P. & Von Haeseler, A. NextGenMap: fast and accurate read mapping in highly polymorphic genomes. *Bioinformatics* **29,** 2790–2791 (2013).

4. Li, H. *et al.* The sequence alignment/map format and SAMtools. *Bioinformatics* **25,** 2078–2079 (2009).

5. Van der Auwera, G. A. *et al.* From FastQ data to high-confidence variant calls: the genome analysis toolkit best practices pipeline. *Current protocols in bioinformatics* **43,** 11–10 (2013).

Supplementary Figure Legends

Supplementary Fig. 1 | Sampling map for source populations and final common-garden. Map area includes Texas and Oklahoma and the locations of the source populations for *H. annuus* ssp. *annuus* (light orange), *H. annuus* ssp. *texanus* (green), *H. debilis* ssp. *cucumerifolius* (pink), and the experimental sites and final common-garden at Lady Bird Johnson Wildflower Center and Brackenridge Field Laboratory (blue).

Supplementary Fig. 2 |

Local gene flow from outside sources into the experimental hybrid population, measured by genome-wide SNPs for LBJ (left panel) and BFL (right panel). (a) The percentage of admixed individuals per generation. Individuals were considered non-admixed if they contained < 0.5% novel alleles. (b) The percentage of variants not found in the BC1 generation for each advanced-generation individual. (c) The standard deviation for the percentage of variants not found in the BC1 generation for each advanced-generation individual.

Supplementary Fig. 3 | Correlations among traits. Raw correlations among traits for controls and hybrids. For hybrids, the upper left values represent the correlations for BFL, and the lower right values represent the correlations for LBJ. Red indicates a positive correlation, blue indicates a negative correlation.

Supplementary Fig. 1

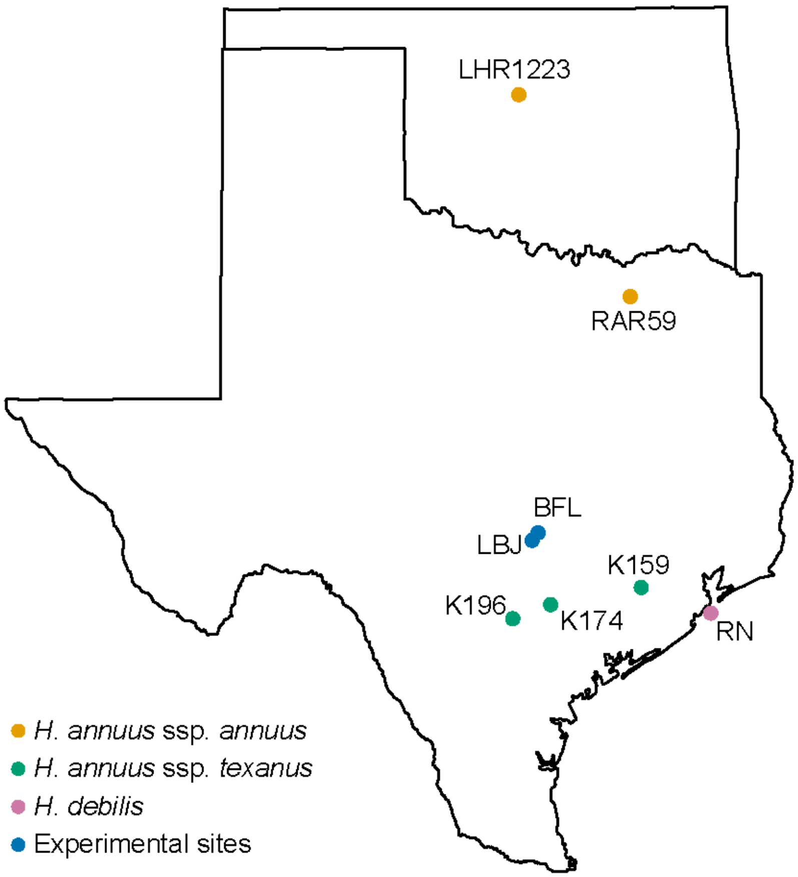

Supplementary Fig. 2

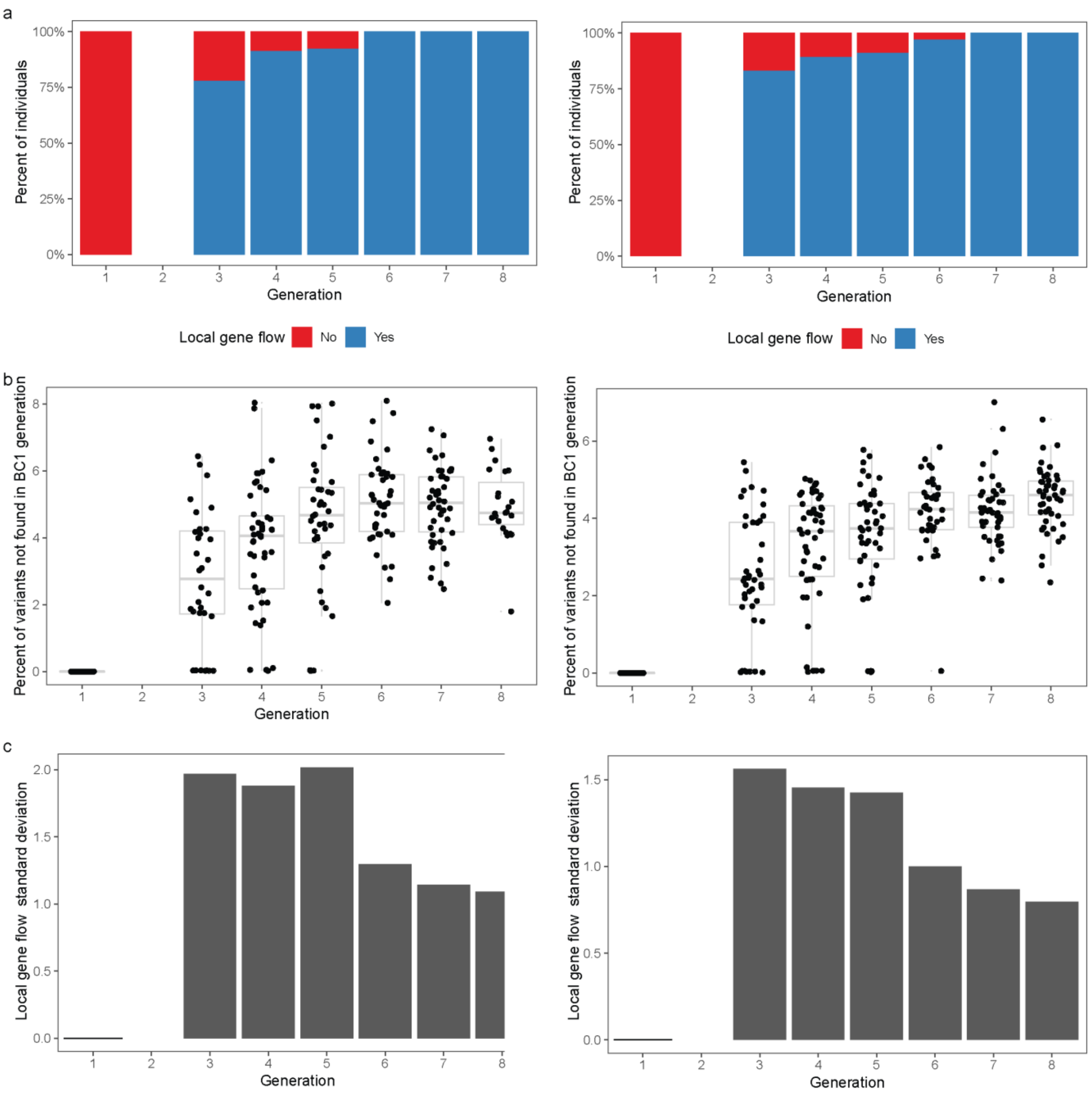

Supplementary Fig. 3

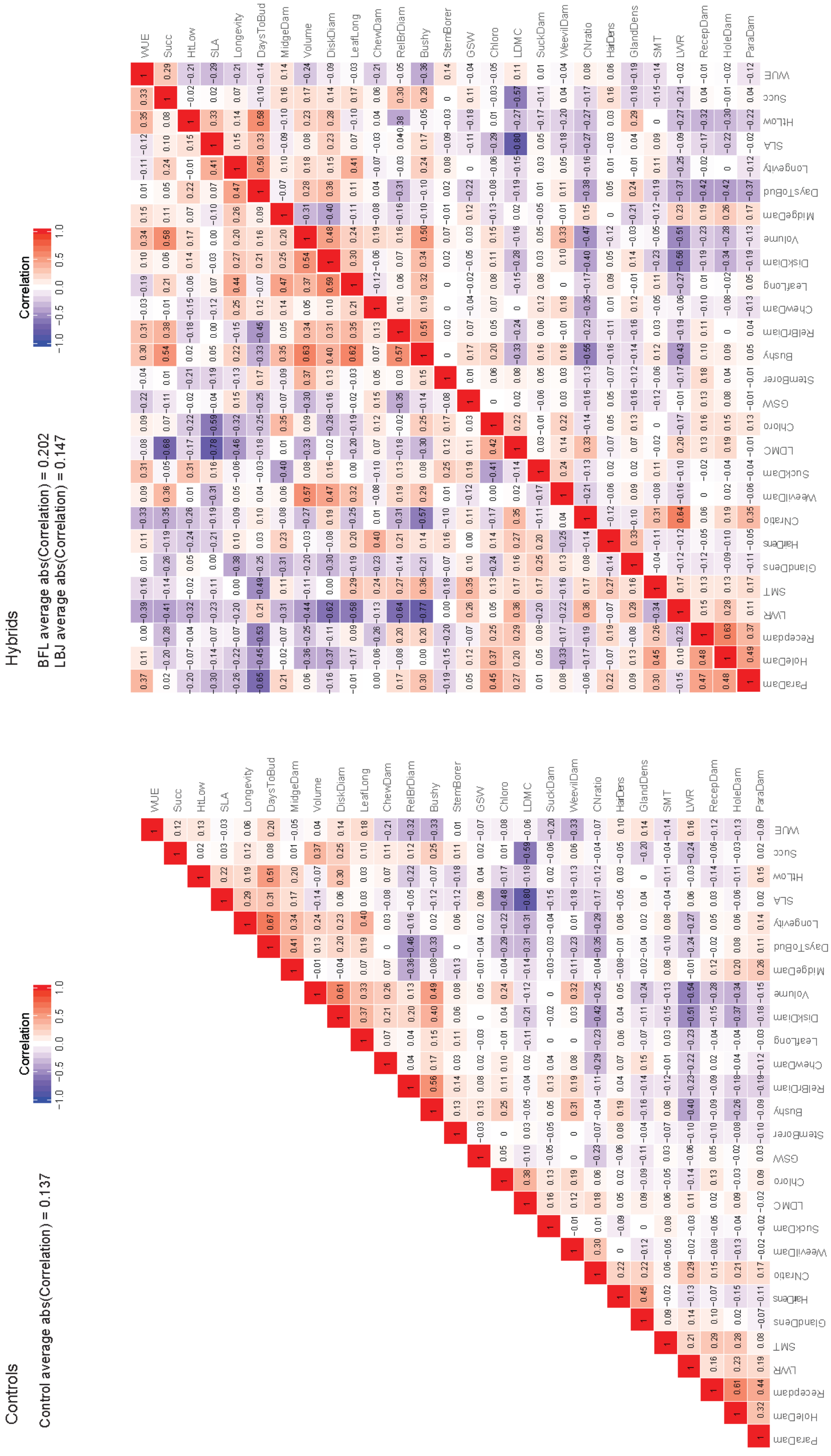

Supplementary Table 1. Trait data summaries.

Trait	Treatment	Generation	N	Mean	sd	se
Fitness	texanus	.	176	2679.638	2975.225	224.266
	Control	1	59	1792.211	1745.273	227.215
		5	28	1570.134	1481.602	279.996
		6	30	1372.406	1388.191	253.448
		7	30	2136.085	1964.703	358.704
		8	59	1750.274	1442.055	187.740
	Hybrid LBJ	1	33	769.117	1012.466	176.248
		5	30	1580.021	1171.189	213.829
		6	29	1872.673	1774.679	329.550
		7	30	2217.428	1365.622	249.327
		8	55	2731.454	2283.980	307.972
	Hybrid BFL	8	30	2000.708	1689.383	308.438
SLA	texanus	.	162	205.832	40.010	3.144
	Control	1	57	183.871	28.033	3.713
		5	27	202.009	41.960	8.075
		6	27	190.940	40.842	7.860
		7	29	181.591	31.139	5.782
		8	55	192.150	32.826	4.426
	Hybrid LBJ	1	32	164.337	29.996	5.303
		5	30	200.095	37.795	6.900
		6	28	185.076	27.433	5.184
		7	30	196.884	36.799	6.719
		8	54	204.320	36.906	5.022
	Hybrid BFL	8	27	193.093	33.979	6.539
LeafLong	texanus	.	160	41.788	5.805	0.459
	Control	1	56	40.786	5.698	0.761
		5	25	41.240	5.472	1.094
		6	28	40.929	6.312	1.193
		7	28	41.393	5.953	1.125
		8	54	40.556	4.316	0.587
	Hybrid LBJ	1	31	40.258	6.366	1.143
		5	28	40.893	3.975	0.751
		6	27	40.481	5.003	0.963
		7	30	40.900	4.374	0.798
		8	53	40.717	4.208	0.578
	Hybrid BFL	8	26	39.846	5.924	1.162
LDMC	texanus	.	162	0.151	0.030	0.002
	Control	1	57	0.158	0.024	0.003
		5	27	0.150	0.030	0.006

		6	27	0.160	0.034	0.007
		7	29	0.168	0.026	0.005
		8	55	0.160	0.032	0.004
	Hybrid LBJ	1	32	0.163	0.027	0.005
		5	30	0.151	0.029	0.005
		6	28	0.160	0.027	0.005
		7	30	0.151	0.028	0.005
		8	54	0.151	0.033	0.005
	Hybrid BFL	8	27	0.150	0.029	0.006
Succ	texanus	.	162	0.029	0.005	0.000
	Control	1	57	0.030	0.003	0.000
		5	27	0.029	0.004	0.001
		6	27	0.029	0.003	0.001
		7	29	0.028	0.004	0.001
		8	55	0.028	0.003	0.000
	Hybrid LBJ	1	32	0.032	0.004	0.001
		5	30	0.029	0.003	0.001
		6	28	0.029	0.003	0.001
		7	30	0.030	0.003	0.001
		8	54	0.029	0.004	0.001
	Hybrid BFL	8	27	0.031	0.004	0.001
Chloro	texanus	.	162	28.007	2.851	0.224
	Control	1	57	32.679	3.256	0.431
		5	27	31.919	3.530	0.679
		6	27	31.889	3.124	0.601
		7	29	31.997	3.637	0.675
		8	55	30.793	3.525	0.475
	Hybrid	1	32	29.678	3.271	0.578
		5	30	29.413	4.037	0.737
		6	28	30.679	2.769	0.523
		7	30	31.490	2.984	0.545
		8	54	30.869	3.501	0.476
LWR	texanus	.	163	1.328	0.193	0.015
	Control	1	57	1.394	0.228	0.030
		5	27	1.373	0.232	0.045
		6	28	1.428	0.223	0.042
		7	30	1.466	0.320	0.058
		8	55	1.359	0.178	0.024
	Hybrid LBJ	1	32	1.575	0.277	0.049
		5	29	1.417	0.224	0.042
		6	28	1.505	0.307	0.058
		7	30	1.363	0.153	0.028

		8	54	1.338	0.208	0.028
	Hybrid BFL	8	28	1.485	0.329	0.062
WUE	texanus	.	30	-30.377	0.576	0.105
	Control	1	30	-30.007	0.644	0.118
		8	30	-30.133	0.652	0.119
	Hybrid LBJ	1	30	-30.113	0.747	0.136
		8	30	-30.490	0.531	0.097
	Hybrid BFL	8	27	-30.511	0.466	0.090
DaysToBud	texanus	.	161	74.957	27.172	2.141
	Control	1	55	58.673	19.137	2.580
		5	27	64.556	20.646	3.973
		6	28	66.071	19.274	3.642
		7	30	54.600	16.819	3.071
		8	54	58.722	13.892	1.891
	Hybrid LBJ	1	32	48.594	12.659	2.238
		5	29	57.448	13.116	2.436
		6	28	52.893	11.269	2.130
		7	29	59.621	14.284	2.652
		8	52	67.481	9.256	1.284
	Hybrid BFL	8	28	51.500	12.315	2.327
SMT	texanus	.	153	20.281	2.923	0.236
	Control	1	47	25.596	4.372	0.638
		5	24	23.875	3.803	0.776
		6	22	23.455	4.103	0.875
		7	24	25.375	5.523	1.127
		8	47	22.085	3.928	0.573
	Hybrid LBJ	1	27	19.222	2.913	0.561
		5	26	21.462	2.775	0.544
		6	25	20.640	3.604	0.721
		7	27	21.111	2.118	0.408
		8	48	20.313	2.918	0.421
	Hybrid BFL	8	27	20.889	3.274	0.630
Longevity	texanus	.	163	180.018	35.725	2.798
	Control	1	56	146.696	28.990	3.874
		5	27	154.852	28.864	5.555
		6	28	149.964	26.014	4.916
		7	30	149.867	30.660	5.598
		8	55	146.327	25.018	3.373
	Hybrid LBJ	1	32	139.563	32.241	5.699
		5	29	138.448	22.687	4.213
		6	28	132.857	20.208	3.819
		7	30	142.800	23.237	4.242

		8	54	147.185	22.503	3.062
	Hybrid BFL	8	28	140.750	24.271	4.587
DiskDiam	texanus	.	161	27.077	4.992	0.393
	Control	1	57	29.068	6.586	0.872
		5	27	27.990	6.547	1.260
		6	27	30.229	6.065	1.167
		7	30	28.506	6.290	1.148
		8	55	28.771	5.442	0.734
	Hybrid LBJ	1	32	23.671	5.900	1.043
		5	29	27.007	6.208	1.153
		6	28	25.559	7.679	1.451
		7	30	29.300	4.712	0.860
		8	54	28.881	5.589	0.761
	Hybrid BFL	8	28	28.256	5.727	1.082
Volume	texanus	.	153	100.178	79.414	6.420
	Control	1	47	77.114	72.349	10.553
		5	24	94.159	89.765	18.323
		6	22	62.983	59.797	12.749
		7	24	95.692	118.652	24.220
		8	47	70.269	62.936	9.180
	Hybrid LBJ	1	25	37.252	36.179	7.236
		5	25	46.046	33.409	6.682
		6	25	54.582	71.923	14.385
		7	26	79.328	66.370	13.016
		8	46	84.057	65.904	9.717
	Hybrid BFL	8	27	68.787	59.695	11.488
HtLow	texanus	.	117	38.256	23.267	2.151
	Control	1	29	38.900	27.129	5.038
		5	16	37.950	23.375	5.844
		6	11	53.027	27.903	8.413
		7	15	28.900	18.147	4.685
		8	36	43.228	25.172	4.195
	Hybrid LBJ	1	17	19.741	13.153	3.190
		5	17	31.682	15.243	3.697
		6	17	29.818	17.528	4.251
		7	19	58.316	29.344	6.732
		8	31	40.990	23.575	4.234
	Hybrid BFL	8	20	22.240	18.843	4.213
Bushy	texanus	.	153	2.335	0.366	0.030
	Control	1	47	2.049	0.334	0.049
		5	24	2.105	0.321	0.065
		6	22	2.033	0.433	0.092

		7	24	1.999	0.410	0.084
		8	47	2.137	0.282	0.041
	Hybrid LBJ	1	25	2.115	0.313	0.063
		5	25	2.152	0.269	0.054
		6	24	2.059	0.380	0.078
		7	26	2.214	0.223	0.044
		8	47	2.224	0.322	0.047
	Hybrid BFL	8	27	2.181	0.377	0.073
RelBrDiam	texanus	.	153	0.271	0.176	0.014
	Control	1	45	0.242	0.206	0.031
		5	24	0.254	0.213	0.043
		6	22	0.174	0.202	0.043
		7	24	0.230	0.207	0.042
		8	47	0.301	0.185	0.027
	Hybrid LBJ	1	25	0.302	0.235	0.047
		5	25	0.298	0.224	0.045
		6	24	0.274	0.213	0.044
		7	26	0.255	0.177	0.035
		8	46	0.226	0.166	0.024
	Hybrid BFL	8	27	0.285	0.186	0.036
GlandDens	texanus	.	162	2023.765	763.338	59.973
	Control	1	57	2177.632	860.501	113.976
		5	27	2322.222	727.196	139.949
		6	27	2212.963	680.864	131.032
		7	29	2160.345	643.772	119.546
		8	54	2448.611	1036.781	141.088
	Hybrid LBJ	1	32	1403.125	682.522	120.654
		5	30	1984.167	770.174	140.614
		6	28	2268.750	991.856	187.443
		7	30	2235.833	795.398	145.219
		8	54	2209.722	1050.386	142.939
	Hybrid BFL	8	27	1775.926	749.983	144.334
HairDens	texanus	.	162	1872.377	522.057	41.017
	Control	1	57	1639.035	424.396	56.213
		5	27	1676.852	442.671	85.192
		6	27	1719.444	404.383	77.824
		7	29	1650.862	423.816	78.701
		8	54	1964.352	556.005	75.663
	Hybrid LBJ	1	32	1777.344	501.785	88.704
		5	30	1805.000	455.777	83.213
		6	28	1950.893	432.557	81.746
		7	30	1868.333	507.314	92.622

		8	54	1805.093	490.124	66.697
	Hybrid BFL	8	27	1775.000	338.052	65.058
CNratio	texanus	.	30	10.980	2.095	0.383
	Control	1	30	10.963	1.213	0.221
		8	30	11.727	1.879	0.343
	Hybrid LBJ	1	30	12.317	2.236	0.408
		8	30	11.097	1.623	0.296
	Hybrid BFL	8	27	12.111	2.014	0.388
SuckDam	texanus	.	155	0.067	0.059	0.005
	Control	1	47	0.123	0.151	0.022
		5	24	0.091	0.073	0.015
		6	23	0.052	0.052	0.011
		7	24	0.051	0.054	0.011
		8	47	0.091	0.106	0.015
	Hybrid LBJ	1	25	0.056	0.069	0.014
		5	25	0.071	0.068	0.014
		6	25	0.087	0.092	0.018
		7	26	0.073	0.072	0.014
		8	47	0.080	0.103	0.015
	Hybrid BFL	8	27	0.076	0.066	0.013
ChewDam	texanus	.	155	0.033	0.035	0.003
	Control	1	47	0.034	0.032	0.005
		5	24	0.039	0.034	0.007
		6	23	0.025	0.020	0.004
		7	24	0.028	0.028	0.006
		8	47	0.039	0.054	0.008
	Hybrid LBJ	1	25	0.013	0.015	0.003
		5	25	0.016	0.014	0.003
		6	25	0.038	0.074	0.015
		7	26	0.019	0.023	0.004
		8	47	0.034	0.035	0.005
	Hybrid BFL	8	27	0.029	0.033	0.006
StemBorer	texanus	.	162	0.253	0.653	0.051
	Control	1	57	0.158	0.649	0.086
		5	27	0.259	0.944	0.182
		6	28	0.214	0.568	0.107
		7	30	0.100	0.403	0.074
		8	55	0.218	0.658	0.089
	Hybrid LBJ	1	32	0.094	0.390	0.069
		5	29	0.103	0.310	0.058
		6	28	0.143	0.356	0.067
		7	29	0.276	0.841	0.156

		8	55	0.182	0.580	0.078
	Hybrid BFL	8	28	0.071	0.378	0.071
WeevilDam	texanus	.	162	0.543	0.940	0.074
	Control	1	57	0.719	1.098	0.145
		5	27	0.444	0.801	0.154
		6	28	0.321	0.723	0.137
		7	30	1.033	1.542	0.282
		8	55	0.582	0.854	0.115
	Hybrid LBJ	1	32	0.375	0.707	0.125
		5	29	0.241	0.577	0.107
		6	28	0.321	0.723	0.137
		7	30	0.367	0.615	0.112
		8	54	0.556	1.003	0.137
	Hybrid BFL	8	28	0.929	1.120	0.212
MidgeDam	texanus	.	161	0.192	0.203	0.016
	Control	1	58	0.304	0.198	0.026
		5	27	0.356	0.234	0.045
		6	27	0.306	0.242	0.047
		7	30	0.235	0.168	0.031
		8	55	0.236	0.139	0.019
	Hybrid LBJ	1	32	0.460	0.203	0.036
		5	29	0.304	0.179	0.033
		6	28	0.198	0.152	0.029
		7	30	0.222	0.134	0.024
		8	53	0.213	0.136	0.019
	Hybrid BFL	8	28	0.292	0.149	0.028
ParaDam	texanus	.	161	0.009	0.020	0.002
	Control	1	58	0.005	0.012	0.002
		5	27	0.005	0.013	0.002
		6	27	0.007	0.012	0.002
		7	30	0.003	0.005	0.001
		8	55	0.003	0.009	0.001
	Hybrid LBJ	1	32	0.006	0.011	0.002
		5	29	0.004	0.008	0.002
		6	28	0.005	0.018	0.003
		7	30	0.004	0.007	0.001
		8	53	0.001	0.002	0.000
	Hybrid BFL	8	28	0.010	0.010	0.002
HoleDam	texanus	.	161	0.047	0.077	0.006
	Control	1	58	0.031	0.051	0.007
		5	27	0.021	0.040	0.008
		6	27	0.015	0.031	0.006

		7	30	0.014	0.020	0.004
		8	55	0.026	0.062	0.008
	Hybrid LBJ	1	32	0.047	0.061	0.011
		5	29	0.017	0.031	0.006
		6	28	0.019	0.050	0.009
		7	30	0.012	0.022	0.004
		8	53	0.007	0.014	0.002
	Hybrid BFL	8	28	0.023	0.031	0.006
GSW	texanus	.	161	0.002	0.006	0.000
	Control	1	58	0.000	0.002	0.000
		5	27	0.000	0.000	0.000
		6	27	0.001	0.004	0.001
		7	30	0.000	0.002	0.000
		8	55	0.002	0.006	0.001
	Hybrid LBJ	1	32	0.008	0.024	0.004
		5	29	0.002	0.009	0.002
		6	28	0.003	0.008	0.001
		7	30	0.001	0.006	0.001
		8	53	0.001	0.003	0.000
	Hybrid BFL	8	28	0.004	0.011	0.002
RecepDam	texanus	.	161	1.639	2.380	0.188
	Control	1	58	1.055	1.210	0.159
		5	27	0.906	1.232	0.237
		6	27	0.751	0.805	0.155
		7	30	0.673	0.727	0.133
		8	55	0.784	1.137	0.153
	Hybrid LBJ	1	32	1.097	1.171	0.207
		5	29	0.622	0.766	0.142
		6	28	0.556	0.728	0.138
		7	30	0.710	0.940	0.172
		8	53	0.452	0.620	0.085
	Hybrid BFL	8	28	1.006	0.994	0.188

For each treatment-generation combination, raw trait data. Number of individuals measured (N), mean trait value, standard deviation (sd), standard error (se). Units correspond to units described in Table 1.

Supplementary Table 2. Posterior comparisons for differences between the control and hybrid standardized fitness values, hybrid - control for different generations.

Experimental Site	Generation	Mean	95% CI	80% CI	Higher fitness
LBJ	1	-0.289*	[-0.672, 0.076]	[-0.535, -0.047]	Control
LBJ	5	0.002	[-0.402, 0.397]	[-0.256, 0.261]	Hybrid
LBJ	6	0.145	[-0.258, 0.552]	[-0.113, 0.412]	Hybrid
LBJ	7	0.022	[-0.377, 0.426]	[-0.234, 0.280]	Hybrid
LBJ	8	0.320**	[0.007, 0.656]	[0.106, 0.541]	Hybrid
BFL	8	0.099	[-0.179, 0.429]	[-0.106, 0.348]	Hybrid

Means, 95%, and 80% credible intervals, as well as which line has the higher fitness according to the mean estimate. * significant at 80% credible level, ** significant at 95% credible level.

Supplementary Table 3. Results from Bayesian regression modeling.

	LBJ					BFL		
	Control		Hybrid		abs(Hybrid) - abs(Control)	Hybrid		abs(Hybrid) - abs(Control)
Trait	Mean	95% CI	Mean	95% CI	Mean	Mean	95% CI	Mean
Fitness	0.001	[-0.047, 0.047]	0.154**	[0.096, 0.211]	0.138**	0.115**	[0.052, 0.180]	0.093**
SLA	0.023	[-0.026, 0.072]	0.146**	[0.089, 0.204]	0.118**	0.116**	[0.048, 0.184]	0.088**
LeafLong	0.000	[-0.051, 0.049]	0.013	[-0.047, 0.073]	0.006	-0.009	[-0.084, 0.067]	0.011
LDMC	0.021	[-0.029, 0.071]	-0.053*	[-0.112, 0.006]	0.028	-0.065*	[-0.137, 0.008]	0.039
Succ	-0.060**	[-0.108, -0.012]	-0.129**	[-0.187, -0.072]	0.069*	-0.056*	[-0.128, 0.017]	-0.002
Chloro	-0.064**	[-0.113, -0.015]	0.060**	[0.003, 0.118]	-0.004	-0.028	[-0.101, 0.047]	-0.027
LWR	-0.002	[-0.051, 0.047]	-0.132**	[-0.190, -0.074]	0.112**	-0.041	[-0.111, 0.032]	0.026
WUE	-0.025	[-0.094, 0.045]	-0.079**	[-0.149, -0.010]	0.045	-0.084**	[-0.155, -0.011]	0.049
DaysToBud	-0.003	[-0.051, 0.045]	0.149**	[0.091, 0.207]	0.129**	0.033	[-0.038, 0.105]	0.020
SMT	-0.092**[a]	[-0.141, -0.044]	0.035	[-0.021, 0.092]	-0.054*	0.073*	[-0.003, 0.148]	-0.019
Longevity	0.004	[-0.046, 0.054]	0.035	[-0.023, 0.094]	0.018	0.006	[-0.065, 0.079]	0.009
DiskDiam	-0.004	[-0.053, 0.044]	0.118**[a]	[0.061, 0.176]	0.098**	0.103**[a]	[0.037, 0.171]	0.083**
Volume	-0.005	[-0.058, 0.049]	0.094**[a]	[0.029, 0.159]	0.072*	0.084**[a]	[0.010, 0.160]	0.063*
HtLow	0.013	[-0.052, 0.079]	0.145**	[0.069, 0.222]	0.117**	0.020	[-0.074, 0.112]	0.013
Bushy	0.020	[-0.034, 0.074]	0.046	[-0.019, 0.111]	0.021	0.026	[-0.051, 0.105]	0.011
RelBrDiam	0.023	[-0.032, 0.077]	-0.052*	[-0.118, 0.013]	0.025	-0.011	[-0.089, 0.068]	0.004
GlandDens	0.030	[-0.019, 0.079]	0.130**	[0.073, 0.187]	0.097**	0.071*	[-0.001, 0.143]	0.039
HairDens	0.070**	[0.021, 0.120]	0.016	[-0.042, 0.075]	-0.043*	-0.001	[-0.075, 0.074]	-0.041
CNratio	0.058*	[-0.011, 0.127]	-0.092**	[-0.160, -0.023]	0.032	-0.013	[-0.088, 0.063]	-0.027
SuckDam	-0.069**	[-0.123, -0.158]	0.033	[-0.031, 0.098]	-0.031	0.042	[-0.036, 0.120]	-0.021
ChewDam	0.002	[-0.052, 0.055]	0.070**	[0.006, 0.135]	0.049*	0.082**	[0.007, 0.158]	0.061*
StemBorer	0.007	[-0.043, 0.057]	0.028	[-0.031, 0.087]	0.012	-0.008	[-0.079, 0.066]	0.009

WeevilDam	-0.009	[-0.058, 0.041]	0.023	[-0.035, 0.083]	0.009	0.081**	[0.013, 0.151]	0.060*
MidgeDam	-0.050**[a]	[-0.097, -0.003]	-0.188**[a]	[-0.244, -0.131]	0.138**	-0.119**[a]	[-0.183, -0.052]	0.072*
ParaDam	-0.015	[-0.064, 0.035]	-0.053*	[-0.112, 0.007]	0.030	0.059*	[-0.010, 0.131]	0.038
HoleDam	-0.030	[-0.079, 0.019]	-0.123**[a]	[-0.181, -0.065]	0.090**	-0.065*	[-0.134, 0.007]	0.034
GSW	0.017	[-0.031, 0.066]	-0.114**	[-0.172, -0.056]	0.090*	-0.026	[-0.097, 0.048]	0.012
RecepDam	-0.046*	[-0.095, 0.003]	-0.083**	[-0.142, -0.024]	0.037	-0.012	[-0.083, 0.062]	0.016

For each trait, posterior means and 95% credible intervals for the regression coefficient between trait value and generation for both control and hybrid lines. The final column (abs(Hybrid) – abs(Control)) refers to the mean for the posterior distribution of the absolute value of the hybrid slope minus the absolute value of the control slope. * significant at 80% credible level, ** significant at 95% credible level, a significant trait evolution in same direction as selection gradient from 2003, indicative of adaptive trait evolution[1,2].

1. Whitney, K. D., Randell, R. A. & Rieseberg, L. H. Adaptive introgression of herbivore resistance traits in the weedy sunflower Helianthus annuus. The American Naturalist 167, 794–807 (2006).

2. Whitney, K. D., Randell, R. A. & Rieseberg, L. H. Adaptive introgression of abiotic tolerance traits in the sunflower Helianthus annuus. New Phytologist 187, 230–239 (2010).

Supplementary Table 4. Selection differentials (s') and selection gradients (β) from 2003.

Controls (n = 460)						
	s'	p-value	*β*	lower	upper	VIF
(Intercept)	.	.	1.00	0.96	1.06	.
SLA	-0.06	0.06	0.02	-0.05	0.10	2.32
Succ	**-0.07**	0.03	-0.06	-0.14	0.01	2.27
LWR	**-0.20**	0.00	0.00	-0.07	0.06	1.55
DaysToBud	**-0.28**	0.00	**-0.12**	-0.19	-0.05	2.16
SMT	0.00	0.92	**-0.07**	-0.12	-0.02	1.11
Longevity	**-0.09**	0.01	0.02	-0.04	0.08	1.62
DiskDiam	**0.21**	0.00	**0.09**	0.03	0.16	1.42
Volume	**0.35**	0.00	**0.27**	0.15	0.38	4.11
Bushy	**0.26**	0.00	**0.14**	0.06	0.21	2.31
RelBrDiam	**-0.10**	0.01	-0.02	-0.10	0.05	2.35
GlandDens	**-0.16**	0.00	-0.03	-0.09	0.02	1.45
HairDens	-0.02	0.65	0.00	-0.06	0.05	1.34
SuckDam	-0.02	0.61	-0.03	-0.07	0.01	1.06
ChewDam	0.04	0.27	0.00	-0.06	0.06	1.12
StemBorer	0.04	0.31	0.01	-0.02	0.04	1.02
MidgeDam	**-0.39**	0.00	**-0.34**	-0.41	-0.27	1.15
HoleDam	**-0.06**	0.01	0.00	-0.06	0.06	1.07
GSW	0.01	0.73	0.01	-0.04	0.05	1.04

LBJ Hybrids (n = 475)						
	s'	p-value	*β*	lower	upper	VIF
(Intercept)	.	.	**1.00**	0.93	1.08	.
SLA	**0.12**	0.04	0.06	-0.06	0.18	3.36
Succ	**0.31**	0.00	0.01	-0.12	0.14	4.70
LWR	**-0.34**	0.00	0.07	-0.02	0.17	2.58
WUE	**0.35**	0.00	0.04	-0.04	0.13	1.44
DaysToBud	**-0.12**	0.03	**0.16**	0.04	0.31	3.66
SMT	**0.27**	0.00	**0.07**	0.00	0.14	1.20
Longevity	**-0.15**	0.01	0.00	-0.07	0.07	1.26
DiskDiam	**0.45**	0.00	**0.10**	0.00	0.20	1.83
Volume	**0.64**	0.00	**0.67**	0.45	0.91	6.91
HtLow	**-0.25**	0.00	**-0.13**	-0.27	-0.02	2.29
Bushy	**0.38**	0.00	**0.18**	0.07	0.29	2.82
RelBrDiam	**-0.41**	0.00	0.12	-0.02	0.24	2.98
GlandDens	-0.06	0.25	0.03	-0.06	0.13	1.45

HairDens	0.06	0.32	0.06	-0.02	0.14	1.35
SuckDam	**0.18**	0.00	0.07	-0.01	0.18	1.09
ChewDam	-0.06	0.25	-0.04	-0.12	0.03	1.11
StemBorer	**0.32**	0.00	**0.18**	0.06	0.45	1.09
CNratio	**-0.35**	0.00	**0.22**	0.11	0.34	2.39
MidgeDam	**-0.63**	0.00	**-0.56**	-0.04	-0.44	1.20
HoleDam	**-0.12**	0.03	**-0.15**	-0.22	-0.09	1.25
GSW	0.09	0.10	-0.06	-0.19	0.08	1.09
RecepDam	**-0.14**	0.02	-0.01	-0.09	0.07	1.49

BFL Hybrids (n = 473)						
	s'	p-value	β	lower	upper	VIF
(Intercept)	.	.	**0.99**	0.94	1.06	.
SLA	**-0.09**	0.03	0.01	-0.14	0.17	4.14
Succ	0.01	0.79	-0.01	-0.18	0.14	3.01
LWR	-0.03	0.45	0.04	-0.01	0.09	1.16
WUE	**0.26**	0.00	0.02	-0.09	0.12	2.49
DaysToBud	0.00	1.00	-0.01	-0.11	0.08	2.47
SMT	**0.15**	0.00	0.04	-0.03	0.10	1.16
Longevity	-0.03	0.50	0.05	-0.02	0.11	1.54
DiskDiam	**0.36**	0.00	**0.23**	0.14	0.31	1.60
Volume	**0.44**	0.00	**0.34**	0.25	0.43	2.26
HtLow	-0.08	0.07	0.02	-0.04	0.10	1.39
Bushy	**0.21**	0.00	**0.18**	0.10	0.27	2.03
RelBrDiam	**-0.09**	0.04	0.06	-0.01	0.14	1.69
GlandDens	**0.15**	0.00	0.05	-0.02	0.13	1.48
HairDens	0.05	0.26	0.03	-0.03	0.09	1.35
SuckDam	**-0.13**	0.00	-0.02	-0.09	0.05	1.28
ChewDam	0.02	0.70	0.03	-0.03	0.09	1.08
StemBorer	**0.12**	0.01	0.06	-0.01	0.15	1.09
CNratio	**-0.13**	0.00	-0.01	-0.09	0.06	1.52
MidgeDam	**-0.34**	0.00	**-0.31**	-0.38	-0.24	1.34
HoleDam	-0.02	0.60	-0.01	-0.05	0.04	1.20
GSW	-0.05	0.20	**-0.04**	-0.10	0.00	1.03
RecepDam	-0.05	0.20	**-0.07**	-0.12	-0.02	1.12

Differentials and gradients in bold are significant at $p < 0.05$ or if 95% confidence intervals (lower and upper) do not overlap zero, respectively. Variance inflation factors (VIF) are calculated from the linear regression model.

Supplementary Table S5. Predicting evolutionary rates.

Control				
Trait	Gen1 mean	TEX mean	TEX - Control1	Evolution slope
SLA	-0.301	0.283	0.584	0.023
LeafLong	-0.057	0.131	0.188	0.000
LDMC	0.095	-0.143	-0.238	0.021
Succ	0.162	-0.106	-0.268	-0.060
Chloro	0.679	-0.611	-1.290	-0.064
LWR	0.038	-0.248	-0.287	-0.002
WUE	0.334	-0.234	-0.568	-0.025
DaysToBud	-0.266	0.515	0.781	-0.003
SMT	0.994	-0.348	-1.343	-0.092
Longevity	-0.159	0.484	0.643	0.004
DiskDiam	0.219	-0.118	-0.336	-0.004
Volume	-0.042	0.238	0.280	-0.005
HtLow	-0.077	0.031	0.108	0.013
Bushy	-0.347	0.423	0.770	0.020
RelBrDiam	-0.044	-0.015	0.028	0.023
GlandDens	0.067	-0.112	-0.179	0.030
HairDens	-0.354	0.117	0.472	0.070
CNratio	-0.239	-0.230	0.009	0.058
SuckDam	0.504	-0.111	-0.615	-0.069
ChewDam	0.133	0.070	-0.063	0.002
StemBorer	-0.067	0.087	0.154	0.007
WeevilDam	0.201	0.014	-0.187	-0.009
MidgeDam	0.266	-0.303	-0.569	-0.050
ParaDam	-0.069	0.268	0.338	-0.015
HoleDam	0.063	0.321	0.258	-0.030

Hybrid					
Trait	Gen1 mean	TEX mean	TEX - BC1	Evolution slope LBJ	Evolution slope BFL
SLA	-0.821	0.283	1.104	0.146	0.116
LeafLong	-0.156	0.131	0.490	0.013	-0.009
LDMC	0.270	-0.143	0.244	-0.053	-0.065
Succ	0.779	-0.106	0.848	-0.129	-0.056
Chloro	-0.150	-0.611	0.295	0.060	-0.028
LWR	0.821	-0.248	0.748	-0.132	-0.041
WUE	0.170	-0.234	0.360	-0.079	-0.084
DaysToBud	-0.717	0.515	1.067	0.149	0.033
SMT	-0.616	-0.348	0.253	0.035	0.073
Longevity	-0.367	0.484	0.893	0.035	0.006
DiskDiam	-0.694	-0.118	0.619	0.11	0.103
Volume	-0.538	0.238	0.907	0.094	0.084
HtLow	-0.759	0.031	0.806	0.145	0.020
Bushy	-0.227	0.423	0.830	0.046	0.026
RelBrDiam	0.269	-0.015	0.026	-0.052	-0.011
GlandDens	-0.833	-0.112	0.701	0.130	0.071
HairDens	-0.075	0.117	0.210	0.016	-0.001
CNratio	0.475	-0.230	0.645	-0.092	-0.013
SuckDam	-0.185	-0.111	0.045	0.033	0.042
ChewDam	-0.439	0.070	0.341	0.070	0.082
StemBorer	-0.171	0.087	0.270	0.028	-0.008
WeevilDam	-0.165	0.014	0.251	0.023	0.081
MidgeDam	1.052	-0.303	1.144	-0.188	-0.119
ParaDam	-0.006	0.268	0.373	-0.053	0.059
HoleDam	-0.025	0.321	0.428	-0.123	-0.065

GSW	-0.178	0.008	0.187	0.017	GSW	0.780	0.008	0.381	-0.114	-0.026
RecepDam	0.014	0.380	0.366	-0.046	RecepDam	0.041	0.380	0.429	-0.083	-0.012

Generation 1 mean standardized trait values for control and hybrids, the mean value for H. a. texanus, the absolute value of the difference between the two, and the absolute value of the mean posterior slope value from the Bayesian regression analyses.

Supplementary Table 6. Population sampling data used in this study.

Species	Population ID	Use	State	Collection Date	Latitude	Longitude
H. annuus annuus	LHR 1223	F_1 parent	Oklahoma	1992	35.90°N	98.10°W
H. annuus annuus	RAR59/SCK21	Recurrent parent	Texas	9/2002	33.31°N	96.24°W
H. annuus annuus	RAR59/SCK21	Control population G1	Texas	9/2002	33.31°N	96.24°W
H. annuus annuus	RAR59/SCK21	Garden	Texas	9/2007	33.31°N	96.24°W
H. annuus texanus	K159	Garden	Texas	10/2005	29.58°N	96.06°W
H. annuus texanus	K174	Garden	Texas	9/2010	29.36°N	97.57°W
H. annuus texanus	K196	Garden	Texas	11/2010	29.18°N	98.20°W
*H. debilis**	RN	F_1 parent	Texas	1998	29.25°N	94.90°W

*GPS coordinates are estimate for Galveston Island.

Supplementary Table 7. Common-garden information.

Species	Home Site	Line	Generation	# Planted	# Analyzed for Architecture	# Analyzed for Isotopes	# Analyzed for Remaining Traits
H. annuus	.	Control	1	60	47	28	57
H. annuus	LBJ	Control	5	30	24	0	27
H. annuus	LBJ	Control	6	30	23	0	28
H. annuus	LBJ	Control	7	30	24	0	30
H. annuus	LBJ	Control	8	60	47	30	55
BC_1	.	Hybrid	1	39	25	25	32
BC_1	LBJ	Hybrid	5	30	25	0	29
BC_1	LBJ	Hybrid	6	30	25	0	28
BC_1	LBJ	Hybrid	7	30	26	0	30
BC_1	LBJ	Hybrid	8	60	47	30	54
BC_1	BFL	Hybrid	8	36	27	27	30
H. a. texanus	wild	K159	wild	60	53	10	53
H. a. texanus	wild	K174	wild	60	49	10	55
H. a. texanus	wild	K196	wild	60	53	10	55

Number of individuals for each line planted in the final common garden, number analyzed for architectural traits (without primary bud damage), and number analyzed for stable isotopes and leaf Carbon:Nitrogen ratio, number analyzed for remaining traits (filtering out early transplant deaths, plants damaged during seed scarification, and plants that lived less than 75 days).
